# Analysis of surgical quality indicators after certification as a Hernia Center

**DOI:** 10.1007/s13304-023-01449-z

**Published:** 2023-02-22

**Authors:** Arnulf Gregor Willms, Sebastian Schaaf, Robert Schwab

**Affiliations:** 1 Department of General, Visceral and Thoracic Surgery, Hernia Reference Center of the German Armed Forces Central Hospital Koblenz, Rübenacher Str. 170, 56072 Koblenz, Germany; 2 Department of General and Visceral Surgery, German Armed Forces Hospital, Lesserstr. 180, 22049 Hamburg, Germany

**Keywords:** Hernia surgery, Incisional hernia, Outcome, Quality management

## Abstract

Certifications are an increasingly used tool of quality management in the health care system. The primary goal is to improve the quality of treatment due to implemented measures based on a defined catalog of criteria and standardization of the treatment processes. However, the extent to which this affects medical and health-economic indicators is unknown. Therefore, the study aims to examine the possible effects of the certification as a Reference Center for Hernia Surgery on the treatment quality and reimbursement dimensions. The observation and recording periods were defined as 3 years before (2013–2015) and 3 years after certification as a "Reference Center for Hernia Surgery" (2016–2018). Possible changes due to the certification were examined based on multidimensional data collection and analysis. In addition, the aspects of structure, process and result quality, and the reimbursement situation were reported. One thousand three hundred and nineteen cases before and one thousand four hundred and three cases after certification were included. After the certification, the patients were older (58.1 ± 16.1 vs. 64.0 ± 16.1 years, *p* < 0.01), had a higher CMI (1.01 vs. 1.06), and a higher ASA score (< III 86.9 vs. 85.5%, *p* < 0.01). The interventions became more complex (e.g., recurrent incisional hernias 0.5% vs. 1.9%, *p* < 0.01). The mean length of hospital stay was significantly reduced for incisional hernias (8.8 ± 5.8 vs. 6.7 ± 4.1 days, *p* < 0.001). The reoperation rate for incisional hernias also decreased significantly from 8.24 to 3.66% (*p* = 0.04). The postoperative complication rate for inguinal hernias was significantly reduced (3.1 vs. 1.1%, *p* = 0.002). The reimbursement of the hernia center increased by 27.6%. There were positive changes in process and outcome quality and reimbursement after the certification, which supports the effectivity of certifications in hernia surgery.

## Introduction

With 25 million operations worldwide and 350,000 procedures per year in Germany for example, hernia surgery represents the quantitatively most significant proportion of interventions in general and visceral surgery departments [1–4]. Thus, hernia surgery has substantial medical and socio-economic relevance [5, 6]. The socio-economic costs of hernia surgery in Germany amount to several billion euros annually [7]. The spectrum of operations ranges from technically simple routine interventions to highly specialized interventions [8, 9]. In the case of the former, inadequacies in the structures or processes harm the quality of the results due to the high number of issues from an individual medical and socio-economic point of view. In the latter case, there is a non-negligible risk for each patient due to the severity of the intervention [8, 9].

In the last two decades, in particular, numerous minimally invasive or hybrid procedures (e.g., (e)MILOS, VAMOS, eTEP, TAPP, IPOM, etc.) [10–12] have been developed to improve the quality of results [13]. However, development always harbors potential risks, so a structured review of the structures, processes, and outcomes is required [14].

Certifications in surgery aim to evaluate and standardize surgical treatment facilities and processes to improve and make quality in the departments transparent. Moreover, since hernia surgery becomes more complex and the spectrum of operative techniques is evolving, there is a need for specialization [15]. To define criteria and requirements that should be met by such centers, the ACCESS Group did so in 2019 with respect to specific differences in European health care systems [15]. Accordingly, such a framework for certification in hernia surgery was already available in Germany for example..

The field of hernia surgery falls within the area of responsibility of the German Society for General and Visceral Surgery e.V. (DGAV e.V.) [16]. The technical requirements in hernia surgery are defined by the CAH (surgical working group on hernia surgery) and the DHG (German Hernia Society) [15, 17].

However, there is hardly any evidence that specialization in hernia surgery leads to better results or outcome. Positive effects are likely, but yet unproven [15, 17].

In addition to the desired positive effects of certification, this is associated with the administrative effort that is not negligible [18]. While the certification effects of entire hospitals have been examined, the certification in hernia surgery has not yet been subjected to a critical examination concerning its effects [19, 20]. The work aims to investigate the possible effects of the certification in hernia surgery to become a reference center on the different dimensions of treatment quality and reimbursement.

## Materials and methods

All patients who underwent hernia surgery in the hopsital´s hernia center between January 1, 2013, and December 31, 2018, were included. The period from 01/01/2013 to 12/31/2015 included the time before certification and from 01/01/2016 to 12/31/2018 the period after certification as an intervention. There were no significant changes in the personnel, especially within the group of consultants. Moreover, the performed operations were standardized by a standard operating procedure (SOP) that defines technical aspects such as technique, mesh type, and fixation.

The hernia center was part of a about 500-bed tertiary referral center in Germany. The surgical department that hosted the hernia center has a capacity of 55 beds. On average, two ORs were managed by the department per day and all hernia patients were treated as in-patients, as no dedicated day-care surgery department existed for hernia surgery. During the study period, the senior surgeons comprised the head of the department and five consultants, whereas four of them were specialized hernia surgeons.

The data were obtained from the documentation of the in-patient stay and the first follow-up examination after 10–14 days as part of the external quality assurance (Herniamed), the HIS (hospital information system), and a query via hospital controlling (CMI, case-mix points, reimbursement data) and by the acquisition of the department's scientific activities and continuing education activities.

Follow-ups were performed standardized for all patients after 10 days and 1 year. The first follow-up after 10 days includes a clinical examination and an ultrasound scan, and if necessary, laboratory findings. At the 1-year follow-up, the patient is contacted in writing with a standardized follow-up questionnaire. If there are any abnormalities, the patient is called in for a clinical and sonographic check-up.

The DGAV GmbH (formerly the SAVC (Service-Organization for General and Visceral Surgery) organizes the certifications as competence and Reference Center for Hernia Surgery for the DGAV as well as the DHG (German Hernia Society).

The requirements for obtaining the certificate relate to the structures, processes, results, and provisions regarding minimum quantities. Quality indicators are also defined. If the requirements are met, an auditor (an experienced hernia surgeon) will check the center as part of an audit. During the initial certification, which is valid for 3 years, the quality assurance structures must be disclosed, among other things. Re-certification is only possible after 3 years if valid data on structural, process, and outcome quality can be presented again. Currently, in Germany, only 12 hospitals are certified as reference centers. In 2016, there were only four in Germany. The following requirements apply concerning external quality assurance for both levels of competence [21, 22]:

- All hernia interventions in adults (≥ 18 years) must be recorded in the Herniamed registry without exception.
- Proof must be provided that follow-up examinations have taken place after 1 year after surgery. These must have taken place over at least 2 months.

The certification regulations stipulate the following minimum requirements for the previously defined quality indicators:

- Total complications of inguinal hernia < 5%.
- Reoperation rate for inguinal hernia within 30 days post-op < 2%.
- Reoperation rate for incisional hernia within 30 days post-op < 10%.
- Infection rate/revision rate for incisional hernia after laparoscopic surgery < 3%.
- Infection rate/revision rate for incisional hernia after open surgery < 10%.

The requirements for obtaining the certification-level Reference Center for Hernia Surgery are as follows according to the certification regulations of the DGAV.

- Minimum volumes: performance of at least 250 hernia operations per year, of which at least 50 must be incisional hernia operations, 5 complex hernias (e.g., parastomal hernia, component separation), and 5 diaphragmatic hernias.
- All requirements for the competence center for hernia surgery must be proven.
- All laparoscopic/endoscopic and open hernia surgery techniques must be available at the reference center.
- Reference centers must conclude a cooperation agreement with a plastic surgeon.
- At least two lectures or posters at DHG-supported or international hernia congresses must be proven annually, or a publication must be published in a peer-reviewed journal.
- A reference center offers continuing education events and job shadowing in the field of hernia surgery. The continuing education events must be certified with eight points per year by the responsible medical association.
- Reference centers must participate in at least one registered multicenter study within 3 years. These studies must correspond to an evidence level 1–3.

Operative standards and surgeons skills were assessed during certification audit by visiting the operation room and observing several operations.

## Data acquisition, aggregation, and statistics

For data acquisition and data aggregation from the different data sources, a data matrix is created in Excel (Microsoft®, Redmont, Washington, USA), which ensures the separate acquisition and processing of the parameters of the two collectives (2013–2015 and 2016–2018). The statistical evaluation uses SPSS (Version 27, IBM, Armonk, New York, USA). Metric variables are always given as mean and standard deviation. Categorical variables are expressed as frequency and percentage.

The data were tested for normal distribution using the Kolmogorov–Smirnov test to meet the requirements for the statistical tests thereafter. Depending On the underlying data scale, the Chi-squared test, or the Student's *t* test was used to examine group differences. The respective *p* value, the odds ratio, and 95% confidence interval are used in the evaluation. The level of significance is assumed to be *p* < 0.05.

## Results

### Description of collectives before and after certification

In the observation period after certification, 1403 patients underwent hernia surgery, which is 6.4% more than before certification (1319 hernia patients). In addition, it is shown that after the certification, the average patient age increased significantly (64 ± 16.16 years vs. 58.11 ± 16.11 years, *p* < 0.01). Another significant difference was found in the ASA score: before the certification, there were significantly more patients in ASA group II (67.3% vs. 61.8%) (*p* < 0.01), while there were more patients after the certification were in the sicker ASA group III (14.1% vs. 12.7%). There is also an apparent increase in the CMI (Case Mix Index). The CMI of the hernia patients increased after certification by 0.0565 points from 1.006 to 1.062, and the sum of the case-mix points of the hernia center per year by 123.801, an increase of 26.1%.

In the 3-year period before and after certification (54.0% vs. 56.8%), surgery on an inguinal hernia is the most common intervention in the hernia center (Table 1); followed by umbilical hernia surgery (29.0% vs. 28.1%), incisional hernia surgery (13.0% vs. 12.0%), primary epigastric hernia surgery (3.0% vs. 2.2%), hiatal hernia surgery (1.8% vs. 1.9%), and parastomal hernia surgery (0.8% vs. 0.8%). There are no significant differences in the number of patients in the different hernia entities.

**Table 1:** Number of treated hernia entities

	Before certification (2013–2015)	After certification (2016–2018)	*p*
Number of patients			
Inguinal hernia total *N* (%)	712 (54.0%)	797 (56.8%)	0.94
Bilateral inguinal hernia *N* (%)	197 (14.9%)	241 (17.2%)	0.84
Umbilical hernia *N* (%)	382 (29.0%)	394 (28.1%)	0.32
Incisional hernia *N* (%)	171 (13.0%)	165 (11.8%)	0.19
Parastomal hernia *N* (%)	11 (0.8%)	11 (0.8%)	0.53
Epigastric hernia *N* (%)	39 (3.0%)	31 (2.2%)	0.13
Hiatal hernia *N* (%)	24 (1.8%)	27 (1.9%)	0.63

The analysis shows a significantly higher proportion of recurrence operations in the subgroup of incisional hernia operations (1.9% after certification vs. 0.5% before certification, *p* < 0.01) (Table 1). The number of inguinal hernia recurrences operated on and the size of the hernia gap in the incisional hernias, on the other hand, do not show any significant difference between the two groups (Table 2). In addition, the number of emergency interventions shows no significant difference; 3.18% of emergency interventions were performed on hernia patients before certification and 2.9% of emergency interventions after certification (*p* = 0.70).

**Table 2:** Complexity of treated hernia entities

	Before certification (2013–2015)	After certification (2016–2018)	*p*
Complexity			
Recurrent inguinal hernia* N* (%)	112 (8.5%)	112 (8.0%)	0.34
Gap size incisional hernia [cm^2^]	38.86 ± 50.89	33.55 ± 45.90	0.31
Recurrent incisional hernia *N* (%)	6 (0.5%)	27 (1.9%)	< 0.01
Emergency surgery *N* (%)	42 (3.2%)	41 (2.9%)	0.70

Moreover, for a referral center of hernia surgery, the continuously improvement and adoption of innovative procedures according to the scientific data and guidelines is important. To address that, before and after the certification were some adjustments of the operative approaches observed. E.g., the proportion of endoscopically treated inguinal hernias increased after the certification, in accordance with the updated guidelines. Also, the preoperative use of botulinum toxin injections was implemented for the treatment of large incisional hernias.

### Process and result quality

The length of hospital stay was determined for the two hernia entities, incisional hernias and inguinal hernias. It did not differ significantly in the case of inguinal hernia operations (Table 3). In contrast, the length of hospital stay for patients with incisional hernia surgery was reduced significantly after certification.

**Table 3:** Hospital length of stay before and after inguinal and incisional hernia patients

	Before certification (2013–2015)	After certification (2016–2018)	*p*
Hospital length of stay inguinal hernias in days (mean ± SD, [median])	1.87 ± 2.15 (1.0)	2.06 ± 2.30 (1.0)	0.10
Hospital length of stay incisional hernias (mean ± SD, [median])	8.80 ± 5.81 (7.5)	6.74 ± 4.13 (6.0)	< 0.01

#### Duration of operation

The duration of the operation did not differ significantly for inguinal hernia operations (63.3 ± 28.9 min vs. 65.2 ± 28.1 min; *p* = 0.23). On the other hand, the duration of the procedure for incisional hernias shows a reduction of 11 min (9%) on average (112.8 ± 69.9 min vs. 123.8 ± 79.5 min; *p* = 0.18).

**Fig. 1:**
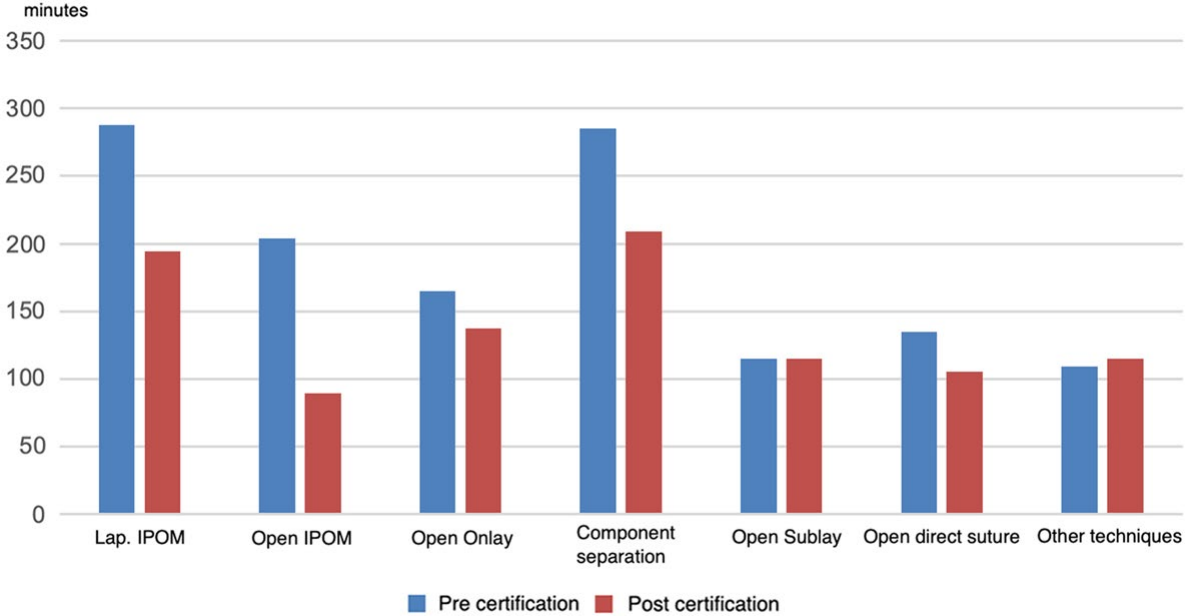
Operation duration in minutes for incisional hernia repair techniques

When considering the duration of operations for incisional hernia operations, the duration of almost every operation could be shortened after certification, although the observed effects were not significant. The most significant reductions result from the procedures laparoscopic IPOM and open IPOM. A medium reduction results from the open onlay and the direct open suture. The sublay operation shows the same time before and after certification with 115 min. Only in the case of the other procedures, under which any other method is subsumed, does the certification show a slightly longer operation time of 6 min on average (Fig. 1).

### Scientific research and training

**Fig. 2:**
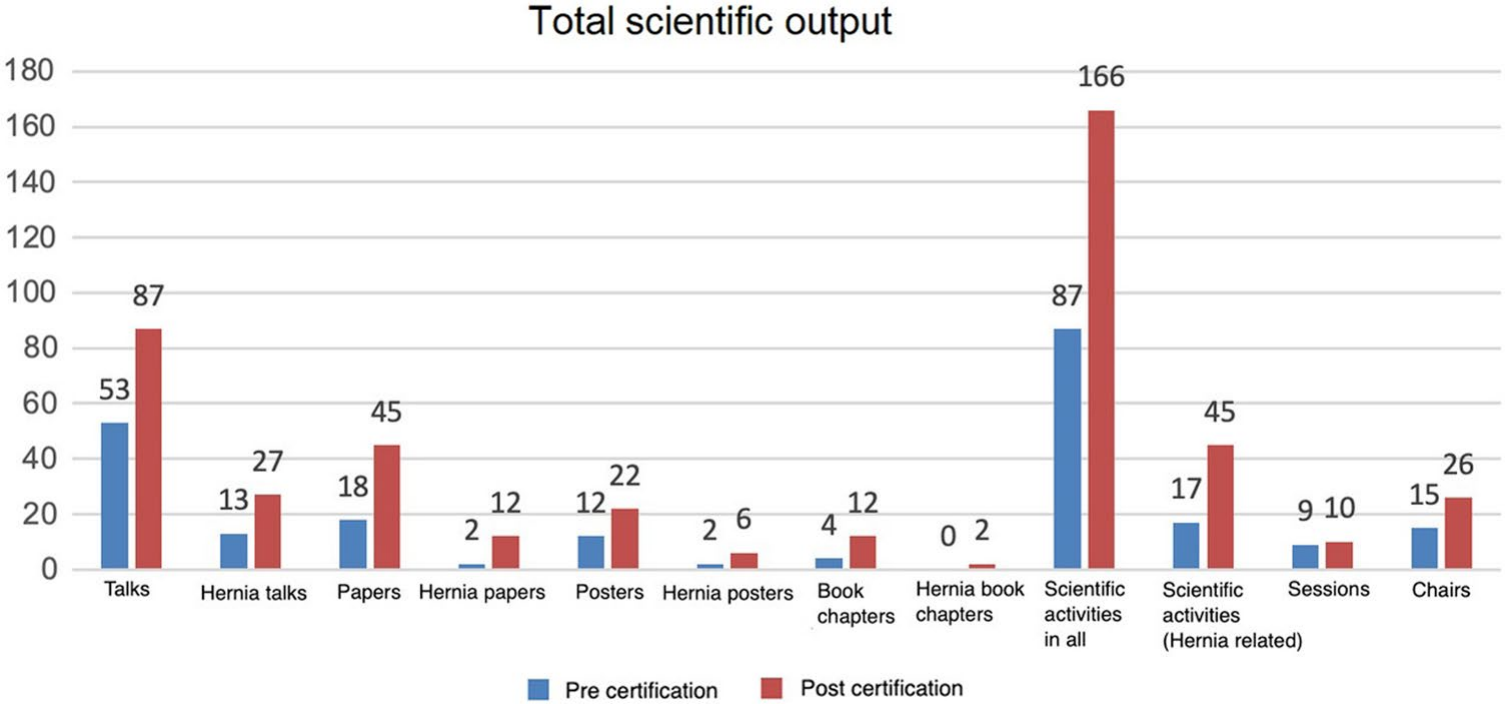
Depiction of research activities before and after certification. The figures show the numbers of different types of scientific publications during the referenced period. The numbers represent the total scientific output of the hernia center

Comparing the two 3-year periods shows an apparent increase in scientific activities. For example, the activities were increased by 84.1% (166 vs. 87) and the services from hernia surgery by 276.5% (45 vs. 17) (Fig. 2).

After certification, the number of CME points awarded to surgeons designated for hernia surgery has increased significantly by 158.6%, more than doubling.

### The outcome of surgical treatment

The mortality rate was very low over the study period and did not change significantly (0.2% before and 0.1% after certification).

The overall follow-up rates were as follows: for 2013–2015, the 10-day follow-up was documented in 96.3% and the 1-year follow-up in 91.4%, whereas the rates were, for 2016–2018, 94.2% (10-day follow-up), and 88.2% (1-year follow-up).

**Fig. 3:**
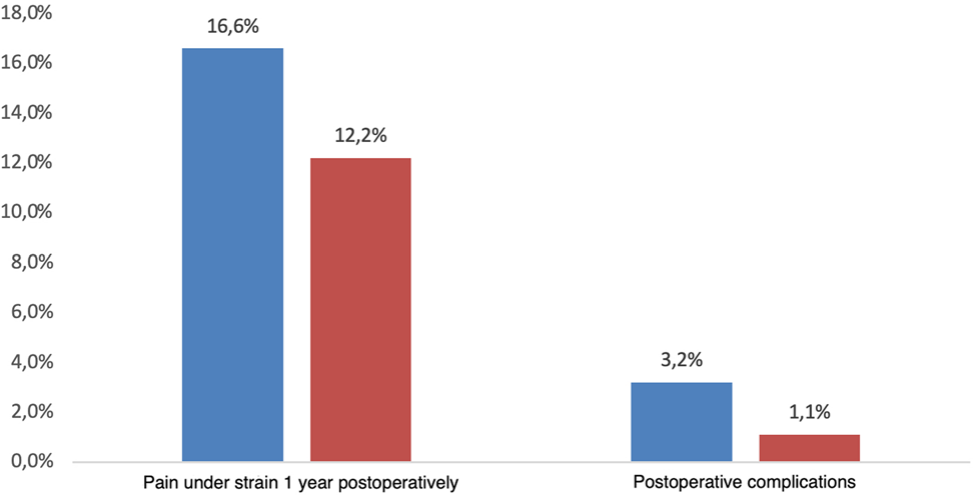
Postoperative complications and pain under strain 1 year after inguinal hernia repair

Postoperative complications after inguinal hernia repair reduced from 3.1% before certification to 1.1% after certification (*p* = 0.002) (Fig. 3). An odds ratio of 0.35 (95% CI 0.18–0.67) corresponds to a 65% risk reduction for postoperative complications. In addition, the rate of postoperative pain decreased from 16.2% to 12.2% after 1 year (*p* = 0.03) (Fig. 3). With an odds ratio of 0.72 (95% CI 0.54–0.96), this means a risk reduction of 28% for the occurrence of postoperative pain after 1 year.

**Fig. 4:**
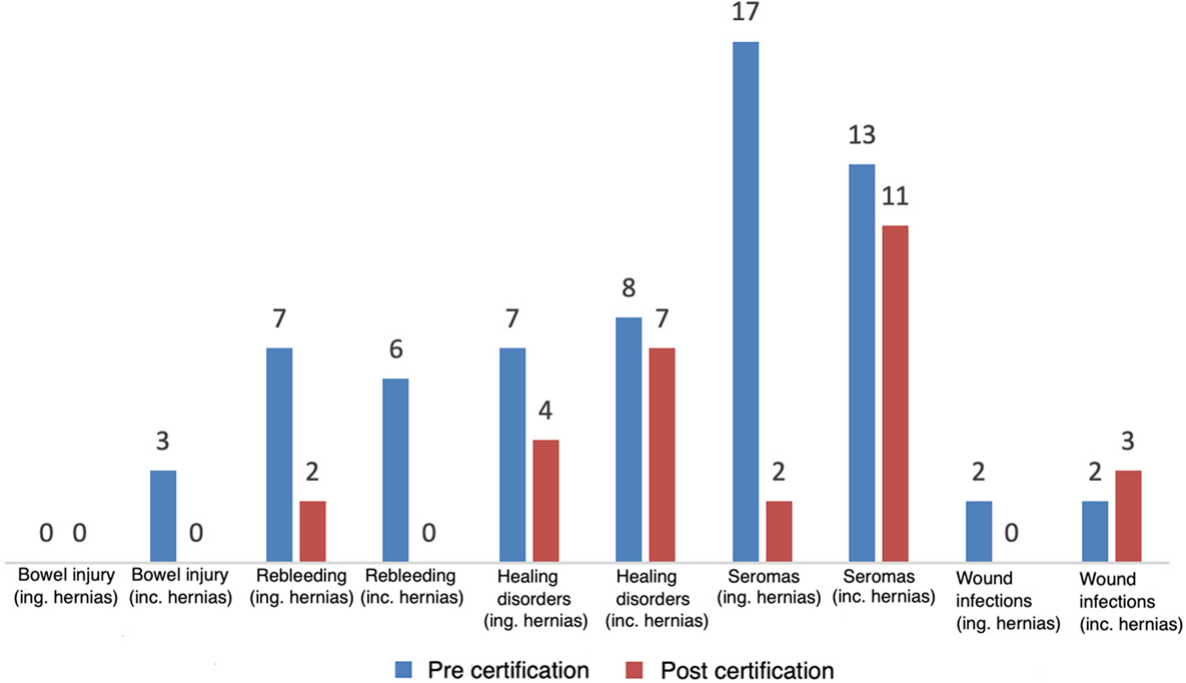
Complication rates before and after certification for inguinal and incisional hernias

While the rate of intraoperative complications showed a slight, non-significant increase from 0.9% to 1.4% (*p* = 0.25), the overall rate of complications also decreased, non-significantly, from 3.9% to 2, 5% (*p* = 0.06) (Fig. 4).

There was no significant difference in terms of recurrence rate (1.1% vs. 1.2%; *p* = 0.82) nor in terms of reoperation rate within 30 days (1.1% vs. 0.6%) (*p* = 0.31) can be detected. A detailed analysis of the postoperative complications shows a reduction in all documented postoperative complications after inguinal hernia surgery, with fewer wound healing disorders, fewer seromas, and less postoperative bleeding. Postoperative infections did not occur 3 years after certification (Fig. 4).

### Incisional hernias

**Fig. 5:**
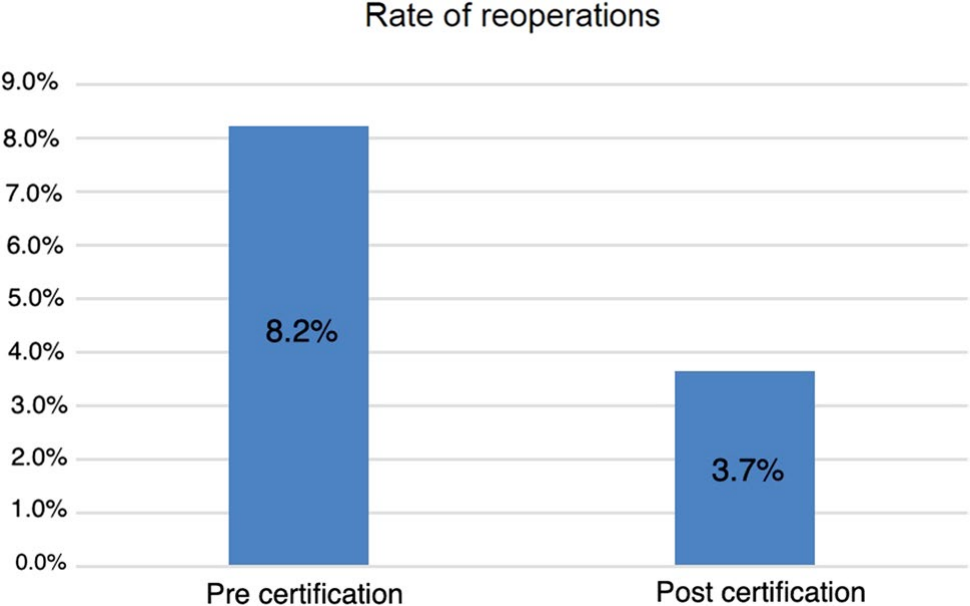
Reoperation rate before and after certification

When comparing the two collectives of the observation periods, a significant difference could be determined for the reoperation rate after incisional hernia repair as one criterion of the quality of the results of incisional hernia surgery. Within 30 days after incisional hernia repair, the reoperation rate decreased from 8.2% to 3.7%; *p* = 0.049 (Fig. 5). That corresponds to a risk reduction of 65% after certification for a complication-related reoperation after incisional hernia surgery.

There was no significant change after certification in any other criteria of postoperative outcome quality after incisional hernia surgery. After incisional hernia surgery, the overall complication rate decreased from 9.3% to 6.04%; *p* = 0.26 (Fig. 4). Except for one infection, which was more frequent after certification than before certification, all complications were reduced. In addition, neither postoperative bleeding nor intestinal injury occurred 3 years after certification.

### Reimbursement

Apart from the national monetary inflation, there are no economic development changes, as medical policy and hospital financing framework conditions have not changed during the examined period, but the reimbursement increased significantly (Table 4). It is noteworthy that reimbursement does not exclude costs and expenses in that number, so it was not the revenue.

**Table 4:** Reimbursement before and after Certification

Measure	Before certification (2013–2015)	After certification (2016–2018)	Difference
Reimbursement (Euro)	1,609,670.04	2,053,648.95	443,978.91 (27.58%)

## Discussion

The present work examines the certification as a Reference Center for Hernia Surgery concerning its associated effects on the different dimensions of surgical treatment quality. Certifications have been increasingly sought in hospitals in recent years [23, 24], even though there is no evidence for their effectiveness [15].

An improvement of quality is considered to be caused by a bundle of measures to be taken and quality management is merely a process than a singular intervention. Thus, certifications are punctual assessments to evaluate whether the process of change has been effective. From the hospitals' point of view, additional objectives are pursued through successful certification. The data on the quality of results obtained in the certification process and through obligatory external quality assurance measures can be used to control and improve one's quality of results [15, 25, 26]. Providing proof of the positive external quality assessment serves as communicable proof of one's quality ,and thus represents a competitive advantage. Certifications are increasingly in demand by informed patients and are also economically relevant to payers since some services are only more successful if certification can be liquidated.

Due to the not inconsiderable use of resources and the commitment of personnel, certifications just for the sake of the certificate should be avoided as far as possible [18, 27, 28]. In addition to some positive voices regarding certification measures, hospitals have critical opinions [29–32]. Likely dissenting voices of the certification measures for the formation of treatment centers cite quality losses across the board, an increase in the burden on the patients, and a possible worsening of the lack of young people due to the higher staffing requirements of the centers [31, 33–35].

The direct proof of positive effects based on substantial evidence is difficult. Individual studies to check the possible impact of certifications were carried out for certifications of entire hospitals (e.g., according to KTQ or DIN EN ISO 9001) and for certifications of individual disease-related treatment centers such as in cardiology, cardiac surgery but also in oncological surgery, and can identify particular possible positive effects show, but paint a rather heterogeneous picture overall [19, 20].

Koeckerling et al. conducted an international survey on the requirements for hernia centers and their surgeons [15]. In addition, 18 European hernia experts were interviewed on critical questions. Essential core requirements for hernia centers for which a consensus could be reached are the following: there will be a higher number of cases, more significant experience of the responsible surgeons, reliable mastery of all open and also laparo-endoscopic hernia interventions, treatment according to current guidelines, recording of all operations in an external registry, and standardized follow-up checks are required [15].

The establishment of a center for hernia surgery must do justice to a particular circumstance since, on the one hand, it has to be offered on a large scale due to the high incidence of hernias. It cannot generally be centralized, but on the other hand, the surgical options and surgical techniques have developed dynamically in recent years and sometimes very much place high demands on the surgeon's indication and technical skills [9, 11, 12, 36, 37]. To a certain extent, this circumstance is met by the different requirements for competence and reference centers and the simplest way of obtaining the DHG seal "certified hernia surgery." For example, operations on complex hernias and diaphragmatic hernias, for which a high level of surgical expertise is required, are only necessary for reference centers [15]. As a special issue, hernia centers, especially such referral centers of special expertise, should take their role and contribute scientific data of new therapy modalities and concepts. In fact, such issues might be the prophylactic implantation of meshes after laparotomies or in emergency ostomy creation [15, 38].

Regardless of the apparent needs and theoretical advantages of certification in hernia surgery, there is still no evidence of associated positive effects on the quality of medical treatment, which should be the primary focus of these efforts [25].

It has been shown that patients’ age and ASA state changed between the observed periods. One explanation for this observation would be that more complex and older patients are more likely to be sent to certified centers by GPs and other hospitals, as they are then trusted with more specialist expertise. But, of course, the study's intention was an observational setting, and causal effects can only be discussed, not approved. Therefore, we added this aspect to the discussion.

Significant changes have been shown above all in the area of result quality as the primary focus of quality management measures [25]. The DGAV defines five quality indicators in the certification scheme, related to avoid complications in both inguinal hernia surgery and incisional hernia surgery [22]. The rate of postoperative complication in inguinal hernias, the rate of exertional pain at 1 year in inguinal hernia patients, and the rate of reoperations in incisional hernias within 30 days improved significantly. The reoperation rate for incisional hernias as another quality indicator relevant to certification and must not exceed 10% fell significantly from 8.2 to 3.7%. The general complications after inguinal hernia surgery also fell from 3.9 to 2.5%, although the significance level was not reached. A long-term parameter in the follow-up also showed a positive development after certification with a significant reduction in pain during exertion after inguinal hernia surgery after 1 year.

A corresponding scientific activity is required in the certification regulations. For example, at least two lectures or posters at DHG-supported or international hernia congresses must be proven, or a publication must be published in a peer-reviewed journal [22]. These requirements are exceeded many times over, especially after certification.

Most recently, the socio-economic aspect of surgical action before and after certification was examined, showing a relevant increase in reimbursement of 27.6% (EUR 443,978.91 in 1 year). In addition to optimizing results, this is an aspect that the center sees as very important. However, a much more extensive analysis would be relevant, taking into account personnel, material, and infrastructure costs. Not all of these factors are clearly defined or partially overlap. In the literature, the focus is usually on clearly definable costs, such as the cost of disposable materials, the length of the operation, and the cost of the in-patient stay. However, variables that are difficult to calculate precisely and, above all, proportionally, such as expenditure on investments, infrastructure, or administration, also play a role.

Other results from the present work that considers economic profitability are the aspects of the length of stay in the hospital and operation times already mentioned above. An essential factor in the cost analysis is the collection of the operation time.

The 63 and 65 min of inguinal hernias operating time shows no difference between the two collectives. The determined times correspond to the times available in the literature, which vary between 32 and 95 min for laparo-endoscopic surgery and between 31 and 67 min for open surgery in prospective randomized controlled studies [39–43]. It should be emphasized here that the intervention in the case of an inguinal hernia in our center is a training intervention, which an assistant doctor carries out under the supervision of a specialist or senior doctor, except for recurrence interventions. Despite increasing complexity, the operating time for incisional hernias was reduced by 11 min. The present analysis results show that the length of stay for inguinal hernias remained unchanged. However, the length of in-patient stay for incisional hernias was reduced significantly from 8.8 days to 6.7 days. These results also show that a vital target criterion of functioning quality management, namely the careful and sensible use of resources after certification, is better met [44].

Certifications set a standard of claims and requirements [15]. The possible consequences are multidimensional and can be differentiated according to the element their change (structures, processes, results) or their orientation (employees, patients, hospital, society, etc.). The results of the present work prove the complexity of the associated changes. These can be confirmed based on the first classification of the processes and outcomes and the interests of the individual stakeholders of a hospital. After the certification process, it shows that positive, multidirectional changes have occurred, which affect not only the patients and optimized quality of care but also all other interest groups.

## Limitations

Another point of criticism can be seen in the selection of the periods. Only 3 years before and 3 years after receiving the certificate were recorded. However, it can be postulated that during the 3 years before receiving the certificate, processes that aimed at improving quality were already initiated with a view to the certification that was then sought. Therefore, it can be assumed that the improvements observed after certification would have been even more significant if a more extended period had been covered.

Also, the improved outcome might be caused partly by the training effect of the surgeons. As there were no significant changes in the responsible personnel (dedicated hernia surgeons among the consultants), we can exclude that external expertise would be accountable. Moreover, most basic surgical procedures (e.g., inguinal hernias, umbilical hernias) have been performed by surgical residents for educational purposes under the consultant's supervision. Thus, there might be a training effect in the personnel during both periods, but from the author's view, it seems unlikely that this effect explains the improved outcome significantly. However, approval of causal factors was neither intended nor possible with an observational study like that, and the results could merely serve as a starting point for further case-controlled evaluations.

Another point concerning the covered periods is that by recording 6 years, only 3 years are after the certification, no real long-term consequences of the certification process can be documented. So it remains impossible to prove whether a substantial and, above all, continuous improvement process was initiated, or whether it is only the result of a short-term project, the lasting effects of which are lacking, also against the background that the data were recorded immediately after the certification. Furthermore, the possible interpretations of the reimbursement situation are also subject to some restrictions since a much more extensive analysis would have had to be carried out to determine the net profits, considering the personnel and material costs [45]. Therefore, the increased reimbursement determined in work would have to be subjected to further analysis and adjustment.

## Conclusion

Ours is the first study to investigate possible changes in the quality of treatment through or after certification in hernia surgery. Remarkably, no quality indicator has deteriorated, but some have improved. The results support the thesis that a good quality control system, which is necessary to obtain certification, helps improve the outcome quality of treatment (here in the field of hernia surgery). Improvements of quality indicators and even reimbursement seem possible. Nevertheless, the effects of certifications are numerous, not well studied and should be further scientifically evaluated—maybe in a systematic manner with future certification processes. Ultimately, the principles and measures associated with the certification must not represent an annoying evil but should be a central element of the maxim for action.
